# Silver Nanocolloids Loaded with Betulinic Acid with Enhanced Antitumor Potential: Physicochemical Characterization and In Vitro Evaluation

**DOI:** 10.3390/nano11010152

**Published:** 2021-01-09

**Authors:** Iulia Pinzaru, Cristian Sarau, Dorina Coricovac, Iasmina Marcovici, Crinela Utescu, Sergiu Tofan, Ramona Amina Popovici, Horatiu Cristian Manea, Ioana E. Pavel, Codruta Soica, Cristina Dehelean

**Affiliations:** 1 Research Center for Pharmaco-Toxicological Evaluations, Faculty of Pharmacy, “Victor Babes” University of Medicine and Pharmacy Timisoara, Eftimie Murgu Square No. 2, RO-300041 Timisoara, Romania; iuliapinzaru@umft.ro (I.P.); iasmina.marcovici@umft.ro (I.M.); codrutasoica@umft.ro (C.S.); cadehelean@umft.ro (C.D.); 2 Faculty of Pharmacy, “Victor Babeș” University of Medicine and Pharmacy Timisoara, Eftimie Murgu Square No. 2, RO-300041 Timisoara, Romania; 3 Faculty of Medicine, “Victor Babeș” University of Medicine and Pharmacy Timisoara, Eftimie Murgu Square No. 2, RO-300041 Timisoara, Romania; csarau@umft.ro (C.S.); crinela.serban@umft.ro (C.U.); 4 Faculty of Dental Medicine, “Victor Babeș” University of Medicine and Pharmacy Timisoara, 9 Revolutiei Bv., 300041 Timisoara, Romania; tofan.sergiu@umft.ro (S.T.); ramona.popovici@umft.ro (R.A.P.); 5 Faculty of Medicine, “Vasile Goldiș” Western University of Arad, 94 Revolutiei Bv., 310025 Arad, Romania; horatiu.manea@umft.ro; 6 Timisoara Municipal Emergency Clinical Hospital, 5 Take Ionescu Bv., 300062 Timisoara, Romania; 7 Department of Chemistry, Wright State University, Dayton, OH 45435-0001, USA; ioana.pavel@wright.edu

**Keywords:** silver nanocolloids, betulinic acid, enhanced antitumor effect, hepatocellular carcinoma, lung carcinoma

## Abstract

Betulinic acid (BA), a natural compound with various health benefits including selective antitumor activity, has a limited applicability in vivo due to its poor water solubility and bioavailability. Thus, this study focused on obtaining a BA nano-sized formulation with improved solubility and enhanced antitumor activity using silver nanocolloids (SilCo and PEG_SilCo) as drug carriers. The synthesis was performed using a chemical method and the physicochemical characterization was achieved applying UV-Vis absorption, transmission electron microscopy (TEM), Raman and photon correlation spectroscopy (PCS). The biological evaluation was conducted on two in vitro experimental models—hepatocellular carcinoma (HepG2) and lung cancer (A549) cell lines. The physicochemical characterization showed the following results: an average hydrodynamic diameter of 32 nm for SilCo_BA and 71 nm for PEG_SilCo_BA, a spherical shape, and a loading capacity of 54.1% for SilCo_BA and 61.9% for PEG_SilCo_BA, respectively. The in vitro assessment revealed a cell type- and time-dependent cytotoxic effect characterized by a decrease in cell viability as follows: (i) SilCo_BA (66.44%) < PEG_SilCo_BA (72.05%) < BA_DMSO (75.30%) in HepG2 cells, and (ii) SilCo_BA (75.28%) < PEG_SilCo_BA (86.80%) < BA_DMSO (87.99%) in A549 cells. The novel silver nanocolloids loaded with BA induced an augmented anticancer effect as compared to BA alone.

## 1. Introduction

The field of nanotechnology has experienced a noticeable development in recent years and gained significant weight in different areas of research [1,2]. Nanotechnology (NT) is defined as the art of manipulating the matter on an atomic and molecular scale to fabricate nanomaterials (NMs) for different purposes [1,2] such as imaging, sensing, targeted drug or gene delivery, tissue engineering, and nanoscale implants [1,3]. NT is directly linked to scientific domains such as physics, chemistry, biology, and medicine [4]. The application of NT in the medical field (according to PubMed database over 100,000 articles were published whereof more than 45,000 in the last 5 years), currently known as “nanomedicine”, revolutionized the treatment, diagnosis, monitoring, and control of several diseases, including cancer [1]. The NMs proposed for cancer therapy were developed in a wide variety of shapes and sizes, with the ultimate purpose of reducing the amount of required drug dose and controlling the release rate. Besides dendrimers, micelles, and liposomes, the polymeric, ceramic, and metallic nanoparticles also became of great interest. Currently, metallic nanoparticles are highly investigated in medicine, mostly due to their significant properties that allow the conjugation with antibodies, ligands, and drugs [5]. 

Silver-based nanoparticles (Sil NPs) have a significant impact across diverse biomedical areas [6]. Their applications are generally divided into diagnostic and therapeutic uses [4]. Sil NPs are excellent antimicrobial agents, biomedical device coatings, drug-delivery carriers, imaging probes, and diagnostic platforms [6]. Silver presents several advantages as it is cost-friendly and abundantly found in natural sources [6]. Moreover, the physical properties of Sil NPs are influenced by their size, distribution, morphological shape, and surface properties which can be modified by diverse synthetic methods, reducing agents, and stabilizers. Owing to their intrinsic cytotoxicity, Sil NPs have been recommended as antibacterial and anticancer agents [6]. The antitumor property of Sil NPs has been demonstrated against various types of cancers, including hepatocellular and lung carcinomas [6] which are the leading cause of death among cancer patients [7,8]. 

Despite considerable advancement being noted in the oncology domain in terms of detection, diagnostic tools, and anticancer therapy, cancer, by its complex and heterogeneous character, remains the most challenging opponent. According to the current global statistics, cancer is expected to become the leading cause of death worldwide in the 21st century [9] and finding alternatives of efficient anticancer agents should be considered a stringent matter. 

Among the various cancer treatments including different surgical procedures, immunotherapy, and radiotherapy, chemotherapy remains the most widely used strategy [10,11,12]. However, chemotherapy difficulties arose from its lack of specificity, inefficacy, high cost, high toxicity, and reported drug resistance [5]. In the recent years, diverse natural compounds that demonstrated a selective cytotoxic effect in cancer cells, and no toxicity in normal cells, were investigated as potential therapeutic options for new cancer treatment regimens [13,14]. Betulinic acid (BA), a lupane-structured pentacyclic triterpene abundantly found in *Betula* species, could be considered a suitable candidate for anticancer therapy based on its wide spectrum of bioactivities such as antitumor, antiangiogenic, immunomodulatory, hepatoprotective, anti-inflammatory, antiviral, antimicrobial, and antioxidant properties [15,16]. Several in vitro and in vivo studies revealed the potent anticancer activity of BA against different types of cancer, including in liver and lung cancers [8,17]. Regardless of its therapeutic benefits especially in the area of chemotherapy, BA exhibits a poor aqueous solubility and pharmacokinetic limitations. Fortunately, recent studies suggested nano-sized delivery platforms as strategic tools to improve its bioavailability and efficacy [10,18,19]. Among the nano-sized formulations proposed for BA enhanced antitumor activity come the following: poly(lactic-co-glycolic acid)-loaded nanoparticles [18,20], chitosan coated iron oxide nanoparticles [21], carboxylic acid-functionalized single-walled carbon nanotubes [22], gelatin-based dual-targeted nanoparticles [23], bovine serum albumin–poly(L-lactic acid) nanoparticle platform [24], PLGA (Poly Lactic-co-Glycolic Acid)−polyethylene glycol (PEG) polymer nanoparticles [25], etc. To the best of our knowledge the association between silver nanoparticles and betulinic acid was not previously discussed, thus it could be considered a novelty that aims to obtain a synergistic biological effect due to the properties of the compounds involved and to counteract the problems related to the solubility of betulinic acid. 

Therefore, the present study was purported at developing uncoated and PEG (polyethylene glycol)-coated silver colloids (SilCo) as suitable nanodrug carriers for BA with enhanced antitumor potential. In addition, the novel silver nanocolloids loaded with BA were characterized from a physicochemical perspective and the antitumor potential was screened using in vitro models of hepatocellular carcinoma (HepG2) and lung cancer (A549) cell lines. 

## 2. Materials and Methods

### 2.1. Reagents

The chemicals used in the study: betulinic acid (purity ≥ 98%), trisodium citrate, sodium lauryl sulphate, silver nitrate, polyethylene glycol (PEG), phosphate buffer (PBS), trypsin-EDTA (Ethylenediaminetetraacetic acid) solution, dimethyl sulfoxide (DMSO), and Alamar blue reagent (resazurin) were purchased from Sigma Aldrich, Merck KgaA and Thermo Fisher Scientific Inc., Waltham, MA, USA. The culture media—Eagle’s Minimum Essential Medium (EMEM—ATCC^®^ 30-2003™) and DMEM (Dulbecco’s Modified Eagle Medium high glucose), and the cell culture supplements—fetal bovine serum and antibiotic mixture (penicillin/streptomycin) were bought from the American Type Culture Collection (ATCC), Sigma Aldrich, Merck KgaA and Thermo Fisher Scientific Inc., Waltham, MA, USA. The reagents used were of analytical and cell culture tested grade.

### 2.2. Cell Lines

The in vitro experimental part of the study was performed using HepG2 (ATCC^®^ HB-8065™) human hepatocellular carcinoma and A549 (ATCC^®^ CCL-185™) human lung carcinoma cell lines acquired from the ATCC as frozen vials. The cells were cultured in specific culture growth media—Eagle’s Minimum Essential Medium (EMEM—ATCC^®^ 30-2003™) and DMEM, respectively, supplemented with 10% fetal bovine serum (FBS) and 1% antibiotic mixture (100 U/mL penicillin/100 µg/mL streptomycin). The experiments were conducted under standard conditions, according to the cell lines’ bank recommendations (incubation in a humidified atmosphere at 37 °C and 5% CO_2_).

### 2.3. Synthesis Method and Physicochemical Evaluation of SilCo_BA

The synthesis procedure was performed according to previously described methods [26,27]. In brief, a mixture of 0.4 mM of trisodium citrate (TC) and 0.5 mM of sodium lauryl sulphate (SLS) was treated with 1 mM of silver nitrate solution for 30 min, at temperature (80 °C) and under continuous stirring. The formation of silver colloids (SilCo) was verified through the appearance of a pale-yellow color. The PEGylated silver colloids (PEG_SilCo) were synthetized by adding an aqueous solution of polyethylene glycol (PEG) to the previously obtained colloidal silver solution under continuous stirring. The samples of interest, silver colloids loaded with betulinic acid (SilCo_BA, PEG_SilCo_BA, respectively), were obtained by treating the colloidal solutions (simple and PEGylated) with a methanolic solution of betulinic acid (final concentration of 0.05 mM). All samples were kept at low temperature (2–4 °C) and were protected from light in dark glass bottles during experiments (i.e., the physicochemical characterization and the toxicological evaluation).

The techniques selected for the physicochemical characterization of the synthesized silver colloids were UV–Vis absorption spectrophotometry (Cary 60 UV–Vis spectrophotometer; 200–800 nm domain) and Raman spectroscopy (LabRam HR 800 system, a confocal Raman microscope—BX41, 100× and 50× Olympus objectives, a He-Ne laser of 15 mW output at 632.8 nm, holographic gratings of 600 and 1800 grooves mm^−1^, a confocal hole set at 300 µm, and a thermoelectrically cooled CCD (charged-coupled device) camera of 1024 × 526 pixels) to confirm the formation of silver colloids, their purity and optical properties s, TEM to determine the size, shape and size distribution of the colloidal nanoparticles, and photon correlation spectroscopy (PCS) (Zetasizer Nano ZS system) in order to obtain data regarding particle sizes and stability. 

### 2.4. Drug Loading Capacity

To obtain drug loading capacity a methanolic solution of betulinic acid (1 mM) was prepared and analyzed spectrophotometrically at 210 nm (absorption maximum). Subsequently, a calibration curve was created through successive dilutions at 0.05, 0.1, 0.15, 0.2, 0.25 and 0.5 mM. To quantify the encapsulation capacity, the metal nanoparticles (10 mL) were redispersed in the solvent (10 mL methanol) and betulinic acid (10 mg) was added under continuous stirring until a clear solution was obtained. The next day, the samples were centrifuged (10,000 rpm, 30 min), the supernatant was separated, and the absorbance was determined (pure betulinic acid absorption maximum around 210 nm). The drug loading capacity and the encapsulation efficiency were calculated using the formulas described in our previous study [27].

### 2.5. Cytotoxicity Evaluation

To determine the cytotoxic potential of the silver nanocolloids (SilCo and PEG_SilCo) and silver nanocolloids loaded with BA (SilCo_BA and PEG_SilCo_BA), the effect of these compounds on cells’ viability and morphology was assessed. Their impact on the viability of HepG2 and A549 cells was determined by the means of Alamar blue assay. A number of 1 × 10^4^ cells/well were seeded in 96-well plates containing 200 µL of EMEM and DMEM culture medium and were treated with 10 µM of test compounds (SilCo, PEG_SilCo, SilCo_BA, PEG_SilCo_BA, and BA_D) for different incubation time points (24 and 48 h). After treatment incubation time, the cells were incubated with 200 µL of fresh medium and 20 µL of Alamar blue solution for 3 h at 37 °C. The absorbance values were measured at 570 and 600 nm using a microplate reader (xMark™ Microplate spectrophotometer, Bio-rad). The percentage of viable cells was calculated according to the formula presented in a previous article [28]. Changes in cells’ morphology at 48 h post-treatment with test compounds (SilCo, PEG_SilCo, SilCo_BA, PEG_SilCo_BA, and BA_DMSO) were monitored in bright field light using the Olympus IX73 inverted microscope (Olympus, Tokyo, Japan). 

### 2.6. Statistical Analysis

The statistical analysis was performed with GraphPad Prism version 8.3.0 for Windows, GraphPad Software, San Diego, CA, USA. The results were expressed as the mean ± standard deviation (SD). The one-way ANOVA statistical test followed by Tukey’s post-test were applied and the differences between the samples (control and treated) were marked with* (*** *p* < 0.001, and **** *p* < 0.0001).

## 3. Results

### 3.1. Physicochemical Characterization of the Obtained Silver Nanocolloids Loaded with BA

**Figure 1:**
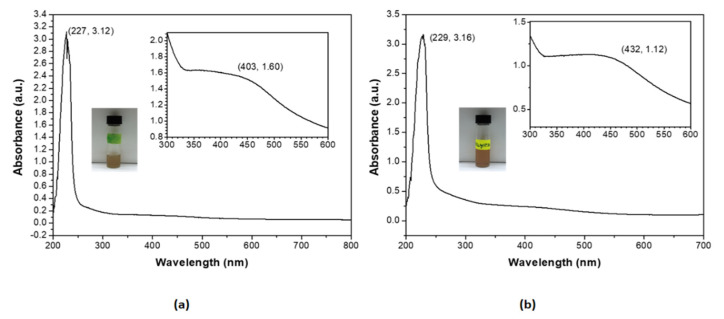
UV–Vis absorbance spectra for the aqueous dilutions (1/10 *v*/*v*) of: (**a**) the silver nanocolloids loaded with betulinic acid and (**b**) the PEGylated silver nanocolloids loaded with betulinic acid. Insets display the aliquot samples of the two silver nanocolloids in glass vials and the expanded spectral areas of the characteristic SPR peaks at 403 and 432 nm, respectively, together with their absorbance values.

**Figure 2:**
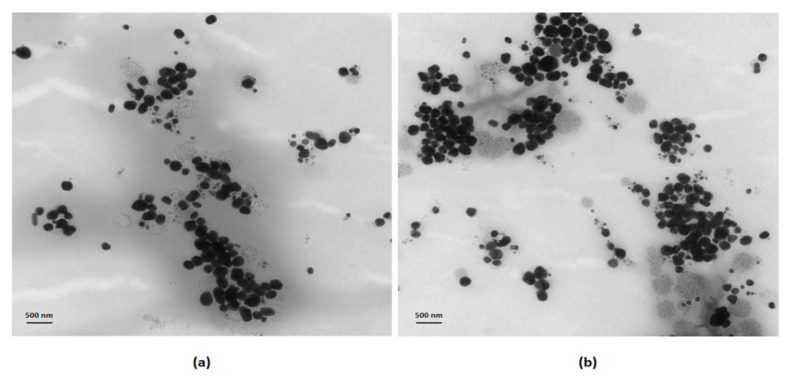
TEM images of: (**a**) the silver nanocolloids loaded with betulinic acid, (**b**) the PEGylated silver nanocolloids loaded with betulinic acid. Scale bars are 500 nm.

The formation of silver nanocolloids loaded with betulinic acid was confirmed by the appearance of pale-yellow color (macroscopic evaluation) and a surface plasmon resonance (SPR) peak in the 400–440 nm spectral range in the UV-Vis absorption spectra (Figure 1). The chemical synthesis was proposed to obtain the desired dimensions and shapes, by using a coating agent with the role of size and stability control (SLS) and a reducing agent (TC). These nanoparticles were in the 20–80 nm size range and had average hydrodynamic dimensions (DLS) of: 21 nm (SilCo), 53 nm (PEG_SilCo), 32 nm (SilCo_BA) and 71 nm (PEG_SilCo_BA). The values of the zeta potentials are presented in Table 1, confirming the stability of the nanoformulations. The spherical shape of the compounds was verified by TEM (HV = 70.0 kV; direct mag 10,000×; 56 Kx) and the corresponding images are shown in Figure 2. The loading capacity of the active substance is important for the transport and distribution of the active substance. In the present study the results are presented in Table 1 showing a good loading capacity of betulinic acid in the proposed formulations.

**Figure 3:**
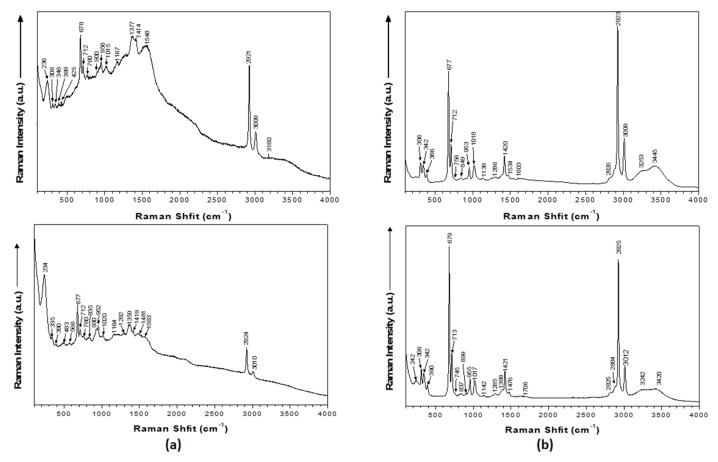
Raman spectra of (**a**) the silver nanocolloids loaded with betulinic acid and (**b**) the PEGylated silver nanocolloids loaded with betulinic acid. The spectra were acquired using 20 s scans and were averaged over 3 cycles.

Modifications in the localized surface plasmon resonance (LSPR) peak profile of silver nanocolloids support the adsorption of betulinic acid to nanosilver’s surface. Characteristic vibrational modes of PEG in the Raman spectrum of the PEG_SilCo_BA sample suggested the PEGylation of SilCo; the peaks attributed to betulinic acid were detected in both SilCo_BA and PEG_SilCo_BA samples at 760, 756, 900, 924, 1015, 1018, 1377 and 1382 cm^−1^. Raman data indicate that betulinic acid is adsorbed onto the surface of colloidal nanoparticles through the carboxy group. The band at 1548 cm^−1^ was attributed to characteristic to the –COO- functional group (Figure 3, Table 2). Moreover, a new Ag-O stretching mode appeared at 236 and 242 cm^−1^. 

### 3.2. Silver-Based Nanocolloids Loaded with Betulinic Acid Induced a Cell Type and Time-Dependent Cytotoxicity

Considering the fact that the novel silver nanocolloids loaded with BA are intended for biological use, their in vitro impact in terms of cell viability and morphology on two tumor cell lines: HepG2—human hepatocellular carcinoma and A549—human lung carcinoma was tested. The results indicated a cell type and time-dependent cytotoxic effect.

**Figure 4:**
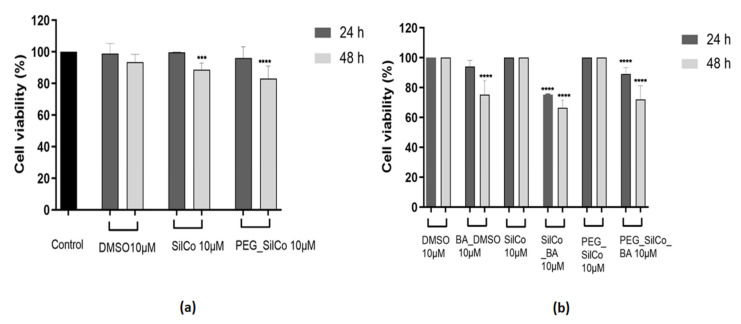
In vitro assessment of silver nanocolloids loaded with betulinic acid effect on human hepatocellular carcinoma (HepG2) cells viability after 24- and 48-h treatment by applying the Alamar blue assay. The data are presented as cell viability percentage (%) normalized to control cells (**a**) and to DMSO, SilCo and PEG_SilCo, respectively (**b**) and are expressed as mean values ± SD of three independent experiments performed in triplicate. To identify the statistical differences between the control and the treated group, one-way ANOVA analysis was conducted, followed by Tukey’s multiple comparisons post-test (*** *p* < 0.001 and **** *p* < 0.0001).

In the case of HepG2 cells (Figure 4a), SilCo and PEG_SilCo had no impact on cells’ viability after a 24 h exposure as compared to the control cells (cells that received no treatment), whereas a longer treatment (48 h) led to a slight decrease in cell viability percentage (approx. 89% for SilCo and 83% for PEG_SilCo, respectively). A similar behavior was observed in the case of DMSO, the solvent used to solubilize BA. Based on these data, the results obtained for silver nanocolloids loaded with BA (SilCo_BA and PEG_SilCo_BA) were normalized to SilCo, PEG_SilCo and DMSO, respectively. Silver nanocolloids loaded with BA treatment induced a decrease in HepG2 cells’ viability percentage that was more significant after 48 h: SilCo_BA (66.44%) and PEG_SilCo_BA (72.05%). A decrease in cells’ viability (75.30%) was also observed in the case of BA solubilized in DMSO (BA_DMSO) but to a lesser extent as compared to the nanocolloids effect (Figure 4b). These results indicate an augmented cytotoxic effect of the novel silver nanocolloids loaded with BA as compared to BA alone. 

**Figure 5:**
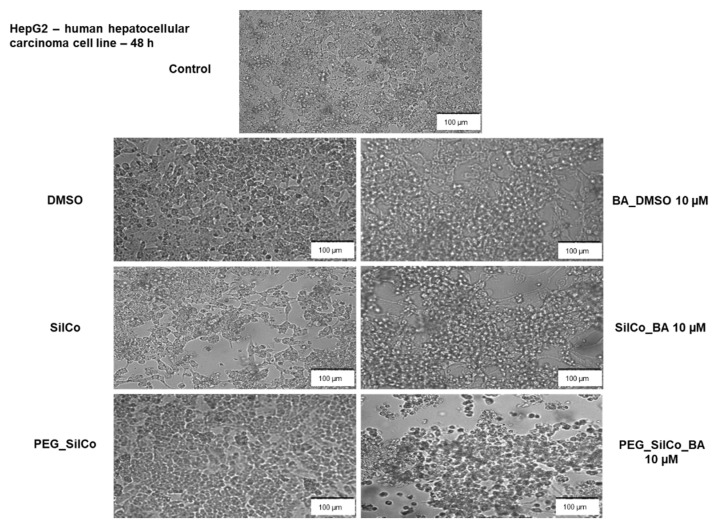
Microscopical appearance of HepG2 cells after a 48-h treatment with 10 µM of test compounds (BA_DMSO, SilCo_BA, PEG_SilCo_BA, SilCo, PEG_SilCo and DMSO). The pictures were taken at 48 h post-treatment under bright field light. Scale bar represents 100 µm.

The treatment of HepG2 cells with 10 µM BA_DMSO and silver nanocolloids loaded with BA (SilCo_BA and PEG_SilCo_BA) for 48 h induced significant changes in cells’ morphology (see Figure 5). A round cell shape, loss of adherence and floating cells in the culture media, and a sporadic distribution of cells were observed when compared to control cells (no treatment). The aspect of DMSO treated cells was very similar to that of the control cells; no significant alterations in HepG2 cells were noticed. The cell exposure to SilCo and PEG_SilCo caused changes in cell shape and adherence, but at a lower rate by comparison with the BA-loaded silver nanocolloids. These alterations in cells’ morphology represent clear signs of cytotoxicity and confirm the cells’ viability assessment results. 

**Figure 6:**
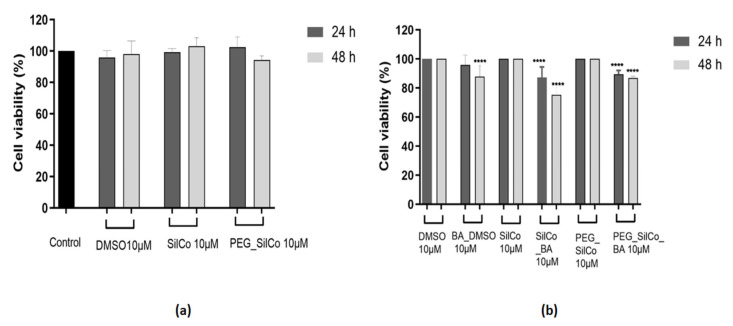
In vitro assessment of silver nanocolloids loaded with betulinic acid effect on A549 cells viability after 24- and 48-h treatment by applying the Alamar blue assay. The data are presented as cell viability percentage (%) normalized to control cells (**a**) and to DMSO, SilCo and PEG_SilCo, respectively (**b**) and are expressed as mean values ± SD of three independent experiments performed in triplicate. To identify the statistical differences between the control and the treated group, one-way ANOVA analysis was conducted, followed by Tukey’s multiple comparisons post-test (**** *p* < 0.0001).

**Figure 7:**
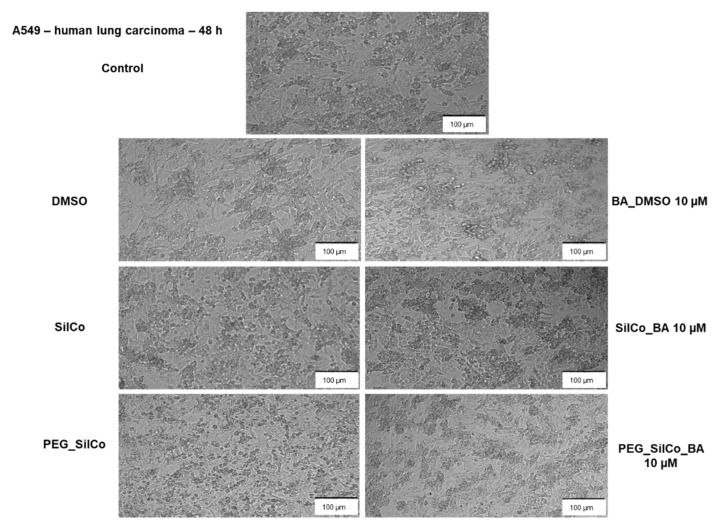
Microscopical appearance of A549 cells after a 48-h treatment with 10 µM of test compounds (BA_DMSO, SilCo_BA, PEG_SilCo_BA, SilCo, PEG_SilCo and DMSO). The pictures were taken at 48 h post-treatment under bright field light. Scale bar represents 100 µm.

SilCo, PEG_SilCo and DMSO did not interfere with A549 cells’ viability; no significant changes were observed in the cell viability percentage after the 48-h treatment (Figure 6a). A549 cells presented a lower susceptibility to BA_DMSO (87.99%) and silver nanocolloids loaded with BA (SilCo_BA—75.28% and PEG_SilCo_BA—86.80%), a decrease in cell viability percentage was recorded but not as significant as for HepG2 cells. Minor morphological alterations were noticed in A549 cells following BA_DMSO and silver nanocolloids loaded with BA treatment (Figure 7). The cells were round, but their adherence was not severely affected and neither their confluency.

## 4. Discussion

Betulinic acid (BA) is a natural compound that was highly investigated due to its potent and selective cytotoxicity oriented toward cancerous cells. The antitumor activity of BA was proved both in vitro and in vivo against various types of cancer such as melanoma, neuroblastoma, breast, colorectal, pancreatic, thyroid, lung, liver cancer, and others [29,30]. BA exerts a multidirectional antitumoral mechanism of action that involves induction of apoptosis (in most tumor cells), inhibition of epithelial-to-mesenchymal transition (in melanoma cells) [31], autophagy, inhibition of angiogenesis, and activation of MAPK pathways [15,16]. The lack of toxicity in healthy cells places BA among the compounds with a wide therapeutic window, a required quality for promising anticancer agents. Besides the plethora of health benefits, BA presents a major inconvenient, a poor solubility in aqueous solutions, which limits its bioavailability and, implicitly, its efficacy in vivo. To overcome this drawback, formulation of BA as a nano-sized system seems a promising option.

In the field of medical and pharmaceutical sciences, the use of nanoparticles (NPs) significantly improved diagnosis and drug-based disease treatments [2,32] by modulating pharmacokinetic and targeting properties of medicines [3]. Nanomaterials can have a positive influence on the therapeutic outcome by adsorbing, binding, and carrying drug molecules to their target [2]. The design of NPs as part of the cancer therapy has given rise to cancer nanomedicine [33] which created a new horizon in future antitumor strategies [34]. In comparison to conventional chemotherapy, nanotechnology provides a novel approach to specifically target cancer cells while avoiding/reducing undesirable side effects [33]. 

At present, silver nanoparticles (Sil NPs) represent one of the most studied metallic nanosystems in terms of biomedical potential due to their exclusive physicochemical (chemical stability, high thermal and electrical conductivity, catalytic activity, and augmented optical characteristics) and biological properties (antibacterial, antiviral, antifungal, anti-inflammatory, and antitumoral effects) [35,36]. These intrinsic features recommend Sil NPs as drug delivery nanoplatforms for antimicrobial, anti-inflammatory, and anticancer compounds [34,36]. 

Based on the previously described anticancer effect of both Sil NPs and betulinic acid, as well as the advantages that drug carriers bring to cancer therapy and the fact that the association between nanoscience and bioactive natural compounds is extremely attractive nowadays [32], this study focused on the preparation of silver nanocolloids loaded with BA of enhanced bioavailability and antitumor activity.

The selection of the synthesis method plays a key role in obtaining optimal drug-delivery platforms for bioactive compounds. It is well known that the physicochemical properties of the NPs such as size, diameter, zeta potential, and coating decide the fate of the novel nanosized system in terms of bioavailability, efficacy, and toxicity [37]. 

Silver-based nanocolloids loaded with betulinic acid synthesized by the described bottom-up chemical approach led to the formation of NPs of spherical shape (Figure 2) and controlled size (Table 1) that does not exert increased toxicity due to the release of silver ions and does not affect cellular integrity [38,39]. This synthesis method proved to be efficient in obtaining stable silver nanoparticles loaded with betulin (a natural compound, member of the same family as BA, the pentacyclic triterpenes) of improved biological properties [27]. Raman data further confirmed the formation of the desired nanocolloids functionalized with BA through the appearance of characteristic vibrational modes: Ag-O and Ag-Cl stretching’s at 236 cm^−1^; COO^-^ bending at 756–760 cm^−1^; CH deformation at 894–901 cm^−1^; rocking vibrations (CH_3_) + rocking vibrations (CH_2_) + twisting (C_16_H_20_H_21_) + bending (CH) at 1015–1022 cm^−1^; CH_2_ and CH_3_ rocking at 1156–1167 cm^−1^; CH_2_ bending and ν_s_(COO) at 1375–1384 cm^−1^; 1534–1548 cm^−1^ –COOH; 1599–1603 cm^−1^ C=C stretch; C=C stretching at 1599–1603 cm^−1^; COO^-^ stretching at 1703–1717 cm^−1^; C-H stretching at 2808–2810 cm^−1^ [40,41,42]. Zeta potential values (Table 1) confirmed the strong electrostatic repulsion between particles responsible for controlling their antiaggregating capacity [26,27,43]. Loading capacity values similar to those reported in the literature [27] are indicative of efficacy of the active substance in biomedical field.

An imperative requirement for any new compound or device for biomedical application is the toxicological screening, mainly in the case of nanomaterials wherefore the safety concerns are still debatable. Hence, in the present study two in vitro experimental models, namely liver (HepG2) and lung (A549) cancer cell lines were employed for the assessment of both toxicological impact and antitumor potential of the novel silver nanocolloids loaded with BA (SilCo_BA and PEG_SilCo_BA, respectively).

Although the silver nanosystems have multiple therapeutic benefits, their toxicity (an issue that still needs thorough investigation) and their reduced stability (high tendency to agglomerate), are matters of concern [3,36]. Previous studies indicated that the toxicity of Sil NPs is dependent on the route of administration, nanoparticles’ size, shape, and dose. Signs of toxicity were observed at different levels such as respiratory, dermal, neurotoxicity, hepatobiliary and reproductive toxicity [37]. 

On this basis, the HepG2 and A549 cell lines were selected for toxicological screening. These experimental models are frequently utilized to evaluate alveolar toxicity (A549—human lung carcinoma cell line) and hepatoxicity (HepG2—human hepatocellular carcinoma cell line) [44]. HepG2 cells are often applied as an in vitro model for hepatotoxicity testing due to their capacity to secrete liver-specific plasmatic proteins, while the expressions of the enzymes responsible for metabolism are rather low [44]. A549 cells are considered a suitable model for alveolar toxicity testing in vitro because of their ability to express metabolizing phase I and phase II enzymes with a similar organization as the ones expressed by the lung tissue [44]. 

According to GLOBOCAN estimates (2018), lung cancer is ranked as the most frequently diagnosed type of cancer (11.6% of total number of cases) with a high mortality rate (18.4% of the total cancer deaths) whereas liver cancer (the sixth most common diagnosed) has a lower mortality rate (8.2%) [9]. Moreover, previous studies showed that both Sil NPs and BA acted as cytotoxic agents on both HepG2 and A549 cells [15,37]. Based on these arguments, HepG2 and A549 cell lines were selected as in vitro models for this study.

The in vitro toxicological screening performed in this study consisted of cell viability assessment and monitorization of changes in cell morphology following the treatment with 10 µM of silver nanocolloids loaded with BA (SilCo_BA and PEG_SilCo_BA), SilCo and PEG_SilCo in comparison with the parent compound BA dissolved in DMSO (BA_DMSO). The concentration (10 µM) was selected based on our previous results that showed a lack of toxicity for Sil NPs and PEG Sil NPs at concentrations lower than 10 µM in healthy cells such as HaCaT, the human immortalized keratinocytes [26]. 

The cell viability results indicated a cell type and time-dependent cytotoxicity as follows: (i) HepG2 cells proved to be more susceptible to the test compounds cytotoxic effects; the most significant decrease in cell viability was recorded at 48 h in the following order: SilCo_BA (66.44%) < PEG_SilCo_BA (72.05%) < BA_DMSO (75.30%) (Figure 4b), and (ii) A549 cells were also affected by the test compounds effects (at 48 h) but at a lower extent as compared to HepG2 cells: SilCo_BA (75.28%) < PEG_SilCo_BA (86.80%) < BA_DMSO (87.99%) (Figure 6b), which indicates an augmented antitumoral effects of BA nano-sized formulation.

Treatment with SilCo and PEG_SilCo induced a decrease in cell viability at 48 h for HepG2 cells (Figure 4a). A549 cell viability was not affected (Figure 6a). The cytotoxic effect of test compounds (SilCo_BA, PEG_SilCo_BA, BA_DMSO, SilCo and PEG_SilCo) was also confirmed by the morphological alterations noticed after the 48-h treatment. These include round cells floating in the culture media, loss of adherence and confluency (Figure 5 and Figure 7) and were more prominent for HepG2 cells.

Sil NPs were reported to have anticancer effects due to their ability to disrupt the mitochondrial respiratory chain, to result in ROS generation, to disrupt the ATP synthesis, and to damage DNA [45,46]. According to a study conducted by Ahmadian et al., Sil NPs showed a potent cytotoxicity on hepatocellular adenocarcinoma (HepG2) cells characterized by a marked cell death (apoptosis using the mitochondrial pathway through ROS production) even after 24 h of incubation. Based on these data, Sil NPs were identified as possible candidates for the treatment of liver hepatocellular carcinoma [46]. Similar results on the HepG2 cell line were reported by Zhu et al. [47]. Another study investigated the antiproliferative effect of Sil NPs on lung cancer cells (H1299). The results revealed a dose-dependent cytotoxicity, as the percentage of viable cells decreased with increasing concentration of Sil NPs. The highest tested concentration (20 μg/mL) increased the percentage of apoptotic cells up to 76.7% ± 7.1% [48]. These data are in agreement with our results.

Liu et al. studied the cytotoxic effect of BA on two hepatocarcinoma cell lines (HepG2 and SMMC-7721) and healthy L-02 liver cells. Their results demonstrated that BA significantly inhibited the proliferation of cancer cells while exhibiting little effect on healthy cells. It was proposed that the cell death occurred through apoptosis, as BA dose-dependently increased BAX and cleaved-caspase-3 protein, and downregulated Bcl-2 protein after 48 h of stimulation [49]. The anticancer effect of BA on hepatocellular carcinoma was also confirmed by Wang et al. [17]. Zhao and colleagues observed the ability of BA-NPs to inhibit proliferation, metastatic ability, and arrest cell cycle in lung cancer cells. Their results indicate that BA-NPs treatment (10 µM) reduced the proliferation of HKULC2, H1299, and H23 cancer cells to 33%, 28%, and 24%, respectively. This treatment also reduced the migration potential of HKULC2 cells [8]. Formulation of BA as poly(lactic-co-glycolic acid)-loaded nanoparticles determined an improved anticancer effect in hepatocellular carcinoma as compared to parent compound BA [18].

## 5. Conclusions

In the present study, a new formulation of BA, as uncoated and PEG-coated SilCo, was successfully obtained and exhibited suitable characteristics such as: size in the 20–80 nm range, spherical shape, negative zeta potential and an optimal average hydrodynamic diameter for nano-sized systems. The novel silver nanocolloids loaded with BA exerted an enhanced cytotoxic effect in both HepG2 and A549 cells as compared to the parent compound BA_DMSO by significantly reducing cell viability and altering their morphological aspect. Thus, this study demonstrates the suitability of uncoated and PEG-coated silver nanocolloids as platforms for the delivery of betulinic acid for enhancing its antitumoral effect due to the synergistic effect provided by BA and silver nanocolloids association. Further studies will be performed in vivo to verify both the safety and the antitumoral efficiency of the novel BA nanoformulation. 

## Figures and Tables

**Table 1:** Specific physicochemical characteristics of the silver and polyethylene glycol (PEG)ylated nanocolloids empty and loaded with betulinic acid.

Sample	Z-Average (nm)	PdI	ZP (mV)	LC (%)
SilCo	21 ± 1.35	0.479 ± 0.2	−36.6 ± 0.05	-
SilCo_BA	32 ± 0.86	0.295 ± 0.08	−28.9 ± 0.02	54.1 ± 0.97
PEG_SilCo	53 ± 2.44	0.399 ± 0.4	−32.7 ± 0.1	-
PEG_SilCo_BA	71± 3.06	0.300 ± 0.1	−23.6 ± 0.04	61.9 ± 3.06

Z-Average—average hydrodynamic dimensions; PdI—polydispersity index; ZP—zeta potential; LC—loading capacity.

**Table 2:** Silver nanocolloids and PEGylated silver nanocolloids empty and loaded with betulinic acid together with their tentative Raman mode assignments (533 nm).

SilCo (cm^−1^)	SilCo_BA (cm^−1^)	PEG_SilCo (cm^−1^)	PEG_SilCo_BA (cm^−1^)
	236 *		242 *
	306		306
	346	345	342
	389		386
	678		677
	712		712
757	760	760	756
		842	849
		894	
929	900		
955	956	955	953
	1015	1062	1018
	1167	1138	1139
		1246	
1298		1285	1286
1380	1359		1388
	1414		1420
	1548 *		1534 *
1638		1636	1603
		1703	
		2892	2826
	2921	2928	2923
	3009		3009
3259		3260	3253
3418		3422	3445

* Betulinic acid attributions.

## Data Availability

The data presented in this study are available on request from the corresponding author.
